# Childhood cancer in Gondar University Hospital, Northwest Ethiopia

**DOI:** 10.1186/s13104-015-1440-1

**Published:** 2015-09-24

**Authors:** Sisay Yifru, Dagnachew Muluye

**Affiliations:** Department of Pediatrics and Child Health, College of Medicine and Health Sciences, University of Gondar, P. O. Box, 196, Gondar, Ethiopia; School of Biomedical and Laboratory Sciences, College of Medicine and Health Sciences, University of Gondar, P. O. Box, 196, Gondar, Ethiopia

**Keywords:** Children, Cancer, Northwest Ethiopia

## Abstract

**Background:**

Childhood cancer becomes a public health problem in developing countries which aggravates the burden of childhood mortality by infectious diseases and malnutrition. In poor countries, the death rate for most pediatric cancers is almost 100 %. This study attempts to determine the magnitude, patterns and trends of pediatric malignancies in the study area which is important in re-evaluating existing services and in improving facilities and patient care.

**Methods:**

A retrospective study of 3 year period were carried out among all children aged below 15 years old admitted into the pediatric wards of Gondar University Hospital, Northwest Ethiopia. The charts of all children aged below 15 years old admitted in the pediatric wards due to cancer were reviewed by using the data collection format. Data were entered and analyzed using SPSS version 20 statistical package.

**Result:**

A total of 71 cancer cases were diagnosed and admitted to the pediatrics ward during the study period. More than two-third of the study subjects 50 (70.4 %) were males. The mean age of study subjects was 7 ± 4 year where majority 26 (36.6 %) of the study subjects were ≥10 years. Of all, 43 (60.6 %) were hematological malignancy followed by Wilms tumor 13 (18.3 %), Neuroblastoma 5 (7 %), Rhabdomyosarcoma 3 (4.2 %), Brain tumor 3 (4.2 %), Hepatoblastoma 2 (2.8 %). More than two-third of cases were found to be concomitantly malnourished being stunted, wasted and under weight. Nearly half of patients had not received chemotherapy and majority of those started chemotherapy did not complete all the treatment cycles. Shortage and absence of safe and affordable chemotherapy drugs were the major reasons for therapy interruption.

**Conclusion:**

The study shows increasing childhood cancer cases over the years. Hematological malignancy takes the leading prevalence followed by Wilms tumor and Neuroblastoma. The majority of cases were also discharged without any clinical change that had the only death option. Therefore, the government and the hospital should give emphasis to establish cancer therapy centers and insure accessibility and affordability of chemotherapy drugs.

## Background

Over the past decades, malignancy was not considered as a public health problem in developing countries, particularly Ethiopia, because of the increased burden of infectious diseases. Although 70 % of pediatric tumors are sensitive to treatment, and survival rates approach 80 % in developed countries, greater than 80 % of affected children in Africa die from their disease [1, 2]. There are total estimates of 120,500 new cancer cases per year in Ethiopia and of approximately 6000 new cases of pediatric cancer each year, according to the clinical record finding in Tikur Anbessa Specialized Hospital. Mortality rates for most pediatric cancers are close to 100 % in developing countries, including Ethiopia [3]. The commonest childhood malignancies encountered includes lymphomas, leukemia, soft tissue sarcomas, osteosarcoma and neuroblastoma [4, 5].

The incidence and type of malignancy vary with age, with a peak in the first 5 years of life and a lower incidence in those aged 8–10 years [6, 7]. The greatest variation in incidence of pediatrics cancers occurs in comparisons of high-income to low income countries and may derive from incomplete ascertainment of pediatric cancer occurrence, different risk factors, or differences in risk among different ethnic or racial population subgroups [8].

Childhood cancer is becoming one of the main public health problem while the service and the attention given to the management of this problem is very less. In Gondar University Hospital, where this study is conducted, there is no separate center for the treatment of cancer and drugs are not available regularly. Therefore, this study is important in re-evaluating existing services and in improving facilities and patient care. Reliable information on the magnitude and patterns of pediatric malignancies helps decision-makers to assess programmatic needs, prioritize interventions and monitor progress. Yet the data are very scarce in Ethiopia, where the need to conduct this study is very important.

## Methods

### Study design, area and period

A retrospective cross-sectional study of the 3 year period from September 2010 to August 2013 were carried out among all children aged below 15 years old admitted in the pediatric wards of Gondar University Hospital, Northwest Ethiopia. Gondar University Hospital is one of the referral hospital in Ethiopia located in the Northern part of the country which serves as a general/referral center for more than 5 million people in the surrounding area and neighboring regions. Yet there is no separate unit for the therapy of cancer patients and the infrastructural services for the treatment of cancer is forgotten.

### Study population and sampling procedures

The case files of all children aged below 15 years old admitted in the pediatric wards due to cancer from September 2010 to August 2013 as recorded in the ward register were reviewed.

### Data collection

The case files/charts of all children aged below 15 years old admitted in the pediatric wards due to cancer were reviewed by using the data collection format. Data extracted from the case files/charts included socio-demographic characteristics, diagnosis, duration of hospitalization and outcomes (death, recovery, referral, unknown). Diagnosis was made based on physicians confirmation by fine needle aspiration cytology (FNAC), bone marrow (BM) aspiration, ultrasound, x-ray and computed tomography (CT) scan.

### Data analysis

Data were cleaned, entered and analyzed using SPSS version 20 statistical package. Descriptive statistics were used to analyze and present the data. Survival analysis of duration of hospitalization of the cases were also employed.

### Ethical considerations

Ethical clearance was obtained from Ethical review committee of University of Gondar. Permission was also obtained from the department of pediatrics and child health. In order to keep confidentiality of any information obtained, the data collection procedure was anonymous.

## Results

A total of 71 malignancy cases were found to be admitted to the pediatrics ward over 3 years from September 2010 to August 2013. More than two-third of the study subjects 50 (70.4 %) were males. The mean age of study subjects was 7 ± 4 years where majority 26 (36.6 %) of the study subjects were **≥**10 years, followed by 5–10 years (Table 1). The majority 63 (88.7 %) of cases were from North and South Gondar Zone. Only 7 (9.9 %) came from Gojam and 1 (1.4 %) from Adigrat, Tigray.

**Table 1:** Sex and age of children with type of malignancy at Gondar University Hospital, Northwest Ethiopia, September 2010 to August 2013

Type of malignancy	Sex		Age				Total
	Female	Male	<1 year	1–5 year	5–10 year	≥10 year	
Hematological malignancy	12 (27.9 %)	31 (72.1 %)	3 (7.0 %)	8 (18.6 %)	11 (25.6 %)	21 (48.8 %)	43 (60.6 %)
Wilms tumor	5 (38.5 %)	8 (61.5 %)	0	9 (69.2 %)	4 (30.8 %)	0	13 (18.3 %)
Neuroblastoma	2 (40.0 %)	3 (60.0 %)	0	2 (40.0 %)	2 (40.0 %)	1 (20.0 %)	5 (7.0 %)
Rhabdomyosarcoma	0	3 (100.0 %)	0	0	1 (33.3 %)	2 (66.7 %)	3 (4.2 %)
Brain tumor	1 (33.3 %)	2 (66.7 %)	0	0	1 (33.3 %)	2 (66.7 %)	3 (4.2 %)
Hepatoblastoma	0	2 (100.0 %)	0	2 (100.0 %)	0	0	2 (2.8 %)
Retinoblastoma	0	1 (100.0 %)	0	0	1 (100.0 %)	0	1 (1.4 %)
Osteosarcoma	1 (100.0 %)	0	0	0	1 (100.0 %)	0	1 (1.4 %)
Total	21 (29.6 %)	50 (70.4 %)	3 (4.2 %)	19 (26.8 %)	23 (32.4 %)	26 (36.6 %)	71 (100 %)

**Fig. 1:**
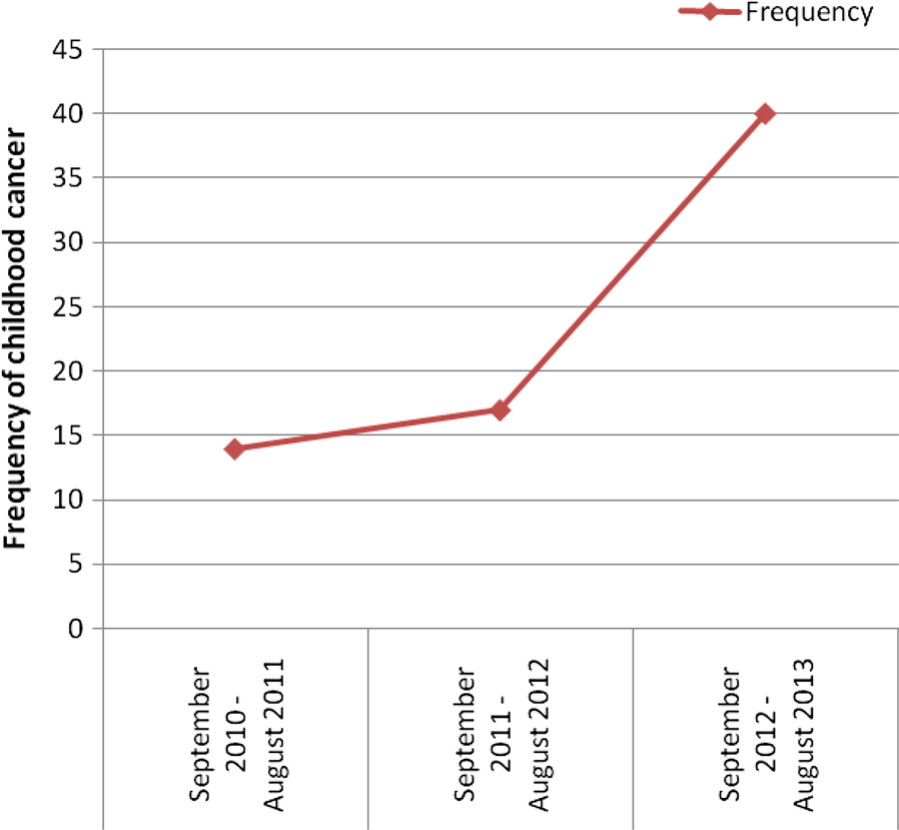
Frequency of childhood cancer per year at Gondar University Hospital, Northwest Ethiopia, September 2010 to August 2013

The frequency of childhood cancer has increased over time (Fig. 1). Of 71 malignant cases diagnosed from September 2010 to August 2013, 43 (60.6 %) were found to have hematological malignancy (13 leukemia, 5 Hodgkin lymphoma, 3 non-Hodgkin lymphoma, 22 unspecified hematological malignancies in the registration) followed by Wilms tumor 13 (18.3 %), Neuroblastoma 5 (7 %), Rhabdomyosarcoma 3 (4.2 %), Brain tumor 3 (4.2 %), Hepatoblastoma 2 (2.8 %). More than 2/3 48 (67.6 %) of cases were found to be concomitantly malnourished being stunted, wasted and under weight. Of 43 hematological malignant cases, 31 (72.1 %) were found among males and higher proportion 8 (61.5 %) of Wilms tumor cases are also among males. Greater than two-third (69.2 %) of Wilms tumor cases were identified from 1 to 5 years age group (Table 1). Five (38.5 %), 1 (7.7 %) and 1 (7.7 %) of Wilms tumor cases were stage 4, stage 3 and stage 2 at diagnosis respectively but rest 6 (46.2 %) were not recorded.

Diagnosis of cases was done primarily based on clinical diagnosis supported by FNAC, BM aspiration, x-ray, ultrasound and CT scan. CT scan was done only for one patient who was having Wilms tumor. Brain tumor was diagnosed only based upon clinical suspicion and x-ray. Thirty-three patients who had hematological malignancy were diagnosed by the support of FNAC and BM aspiration. Ultrasound was done for 12 patients of these 10 were Wilms tumor cases and 2 were Neuroblastoma.

**Fig. 2:**
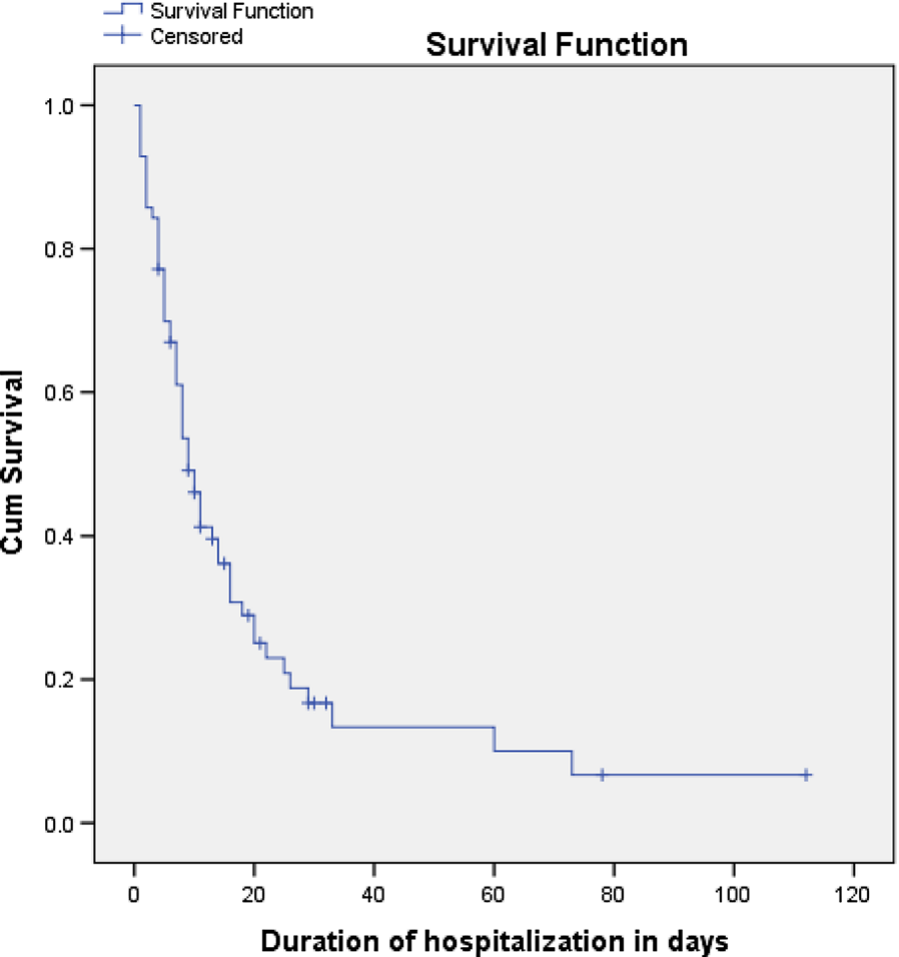
Kaplan Meier survival curve of duration of hospitalization among childhood cancer patients at Gondar University Hospital, Northwest Ethiopia, September 2010 to August 2013

The duration of hospitalization ranges from 1 day to 112 days with a median survival time of 9 days. As shown in the survival curve, Fig. 2, almost all will be out by 120 days with poor prognosis. Thirty-eight (53.5 %) of cases had received chemotherapy but 33 (46.5 %) had not. The majority of cases who started chemotherapy did not complete all the treatment cycles because of many reasons. Three of the referred cases were referred for chemotherapy. The main reasons for most cases that were discharged with no clinical change, chemotherapy interruption and against medical advice are unavailability and unaffordability of the chemotherapy drugs. The majority 46 (64.8) of cases were discharged without any clinical change who had the only death option. All of the deaths are due to Hematological malignancy and 80 % of them died before getting chemotherapy (Table 2).

**Table 2:** Type of malignancy and condition at discharge of children at Gondar University Hospital, Northwest Ethiopia, September 2010 to August 2013

Type of malignancy	Condition at discharge					Total
	Death	Improved	Referred	No clinical change and/or against medical advice^a^	Unknown	
Hematological malignancy	5 (11.6 %)	9 (20.9 %)	3 (7 %)	23 (53.5 %)	3 (7 %)	43 (60.6 %)
Wilms tumor	0	1 (7.7 %)	0	12 (92.3 %)	0	13 (18.3 %)
Neuroblastoma	0	0	0	5 (100 %)	0	5 (7.0 %)
Rhabdomyosarcoma	0	3 (100 %)	0	0	0	3 (4.2 %)
Brain tumor	0	0	0	3 (100 %)	0	3 (4.2 %)
Hepatoblastoma	0	0	0	2 (100 %)	0	2 (2.8 %)
Retinoblastoma	0	1 (100 %)	0	0	0	1 (1.4 %)
Osteosarcoma	0	0	0	1 (100 %)	0	1 (1.4 %)
Total	5 (7.0 %)	14 (19.7 %)	3 (4.2 %)	46 (64.8 %)	3 (4.2 %)	71 (100 %)

^a^No clinical change and/or against medical advice: when the patient does not get improvement and losing hope on the curement of the case, parents resist the physicians advice and discontinue their medical care

## Discussion

Mortality rates for most pediatric cancers are close to 100 % in developing countries, including Ethiopia [3]. A total of 71 cancer cases were diagnosed and admitted to the pediatrics ward over 3 years from September 2010 to August 2013. More than two-third (70.4 %) of the cases were males, which is in agreement with several studies showed male predominance [9–15]. The majority 26 (36.6 %) of the study subjects were ≥10 years in this study while other studies reported among 5–10 age groups [10, 12].

The frequency of childhood cancer is alarmingly increasing over years in this study. It could be due to the increased public awareness and the health seeking behavior of the community. Hematological malignancy takes the leading prevalence followed by Wilms tumor and Neuroblastoma as most of the previous studies notified [9–14]. Of 43 hematological malignant cases, 31 (72.1 %) were found among males and higher proportion 8 (61.5 %) of Wilms tumor cases are also among males which is consistent with the study conducted in Kenya [16].

The majority (67.6 %) of cases were found to be concomitantly malnourished being stunted, wasted and under weight. Previous studies also suggested that children with cancer can experience malnutrition [17, 18]. Malnutrition in children with cancer could appear because of the advanced and the aggressiveness of the tumor. Malnutrition reduces effectiveness of chemotherapy by increasing the length of time and increases likelihood of developing infections 19].

Five (38.5 %) of Wilms tumor cases were stage 4 at diagnosis. This indicates that most cancer cases have come to the health institution lately. Thirty-three (46.5 %) of cases had not received chemotherapy, which needs urgent response from low level to high level professionals and governmental bodies. Unless community awareness is made in line with accessibility of affordable cancer therapy and services, the mortality rate of cancer patients is dangerously high.

The duration of hospitalization ranges from 1 to 112 days with a median survival time of 9 days. Waiting under supportive treatment because of lack of anti-cancer drugs and delayed diagnosis are the main reasons for prolonged hospitalization. Hence, prolonged hospitalization is a gear for cancer treatment by exposing patients for hospital acquired infections. All of the deaths are due to Hematological malignancy. The majority (64.8 %) of cases were also discharged without any change that had the only death option. This shows the lesser attention given for the problem. The majority of cases who started chemotherapy didn’t complete all the treatment cycles because of many reasons. The main reasons for most cases that were discharged with no change, chemotherapy interruption and against medical advice are; shortage and absence of safe and affordable chemotherapy drugs. The Ethiopian government gave focus and working on prevention and control of infectious diseases where cancer cases did not get attention.

In general, critical shortage of anti-cancer drugs in the nation, very late presentation of patients, use of old diagnostic methods and shortage of professionals are aggravating the magnitude cancer. Therefore, cancer therapy centers with full setup are needed to be established in different corners of the country, especially in the study area, Gondar university hospital, which is the only referral hospital in North West Ethiopia serving a population of more than 5 million coming from different geographical locations surrounding it.

## Conclusion

The frequency of childhood cancer is alarmingly increasing over years in this study showing a male predominance. Hematological malignancy takes the leading prevalence followed by Wilms tumor and Neuroblastoma. The majority of cases were found to be concomitantly malnourished being stunted, wasted and under weight. Almost 2/3 of cases were also discharged without any change that had the only death option. The majority of cases who started chemotherapy didn’t complete all the treatment cycles because of shortages and absence of safe and affordable chemotherapy drugs. Therefore, the government and the hospital should give emphasis to establish cancer therapy centers and insure accessibility and affordability of chemotherapy drugs.

## Abbreviations

BM: bone marrow
CT: computed tomography
FNAC: fine needle aspiration cytology
SPSS: statistical packages for social sciences
